# The clinical psychology of ageing in Italy: Priorities for supporting the wellbeing of an ageing population

**DOI:** 10.1371/journal.pmen.0000241

**Published:** 2025-03-18

**Authors:** Giovanni Ottoboni, Elisa Di Rosa, Davide Maria Cammisuli, Maria Casagrande, Gianluca Castelnuovo, Ilaria Chirico, Anna Della Vedova, Isabella Franzoi, Mario Fulcheri, Antonella Granieri, Anna Pecchinenda, Luciano Peirone, Donatella Petretto, Maria Catena Quattropani, Alberto Sardella, Rabih Chattat

**Affiliations:** 1 Department of Psychology “R Canestrari”, University of Bologna, Bologma, Italy; 2 Department of General Psychology, University of Padova, Pavova, Italy; 3 Department of Psychology, Catholic University, Milan, Italy; 4 Department of Dynamic and Clinical Psychology, University of Rome La Sapienza, Roma, Italy,; 5 Psychology Research Laboratory, Istituto Auxologico Italiano IRCCS, Milan and Piancavallo, Italy; 6 Department of Clinical and Experimental Sciences, University of Brescia, Brescia, Italy,; 7 Department of Psychology, University of Turin, Torino, Italy,; 8 Fondazione UNITRE Torino, Torino, Italy; 9 Department of Pedagogy, Psychology, Philosophy, University of Cagliari, Cagliari, Italy; 10 Department of Educational Science, University of Catania, Catania, Italy; PLOS: Public Library of Science, UNITED KINGDOM OF GREAT BRITAIN AND NORTHERN IRELAND

For the first time in history, most of the world’s population can expect to live beyond 65 [1]. The goal has been achieved thanks to improved social and health conditions in the last two centuries. This new course has decreased the number and types of pathogens causing deathly infections; on the other hand, the increase in life expectancy still comes with age-related health problems. Hearing and vision impairments, diabetes, osteoarthritis and osteoporosis, cardiovascular pathologies, and neurodegenerative pathologies are all examples of non-communicable diseases chronically affecting an increasing number of older adults. In this horizon, the burden of the resultant age-related chronic problems would impact national welfare and health systems: looking at demographic and epidemiological data from a perspective that considers ageing as a product of time and inflammation, life expectancy would imply that it is primarily a medical target. Differently, ageing represents, as any other life phase, a multifaceted process featuring adaptations, continuity, learning experiences, and evolution in roles and relationships. Defining the professionals who will care psychologically for ageing people is essential within such scenarios [2]

In Italy, people over 65 are expected to represent 34% of the population between 2045 and 2050 [3], with an incidental rate of ageing-related problems of 4.8 per 10,000 inhabitants and an increased ratio of difficulties in accessing health services [4–6]. Such a demographic revolution has urged the development of a national law inviting local authorities to implement psycho-social care for the ageing population in health and social services [7]. In this line, any act aimed to promote the psychological focus within public, academic, and service-related stakeholders involved in the ageing of the Italian population is crucial [2]. Thus, in this Opinion, we outline the state of the art in this arena and the need for progress in the clinical psychology of ageing

In Italy, the 56/89 law instituted the State regulation of psychological and psychotherapeutic acts [8]. The law defines the profession of the psychologist as any psychological activities directed to prevention, diagnosis, empowerment/ rehabilitation, assistance, and compensation towards individuals, groups, social organisations, and communities. Psychologists can operate in the public or the private sector. Psychologists must possess a psychotherapeutic degree to serve permanently in the public health sector. In this sector, some psychologists work even without the degree mentioned above, but temporarily. The psychotherapeutic degree is supplementary in the welfare public sector, and its possession does not represent an inclusion criterion. In the private sector, psychological services are regulated by the job market. The public health sector can barely offer psychotherapy paths (4), as it primarily focuses on diagnostic assessments or cognate goals. Generally, the interventions provided in the public sector consisted of brief strategic therapy, cognitive-behaviour therapy, eye-movement desensitisation and reprocessing, or focused mindfulness. The psychological support in the public welfare sector is fixed-term, while, as before, the private sector is market-driven

When the psychological profession deploys in ageing contexts, it takes care of any factor generating discomfort in people, such as bereavement, losing skills, abilities, and independence from a psychological point of view. Moreover, it supports people coping with life transitions, continuity, and adaptations. Finally, it provides care for the multiple challenges evoked by chronic diseases. In other words, the clinical psychology of ageing attempts to maximise well-being and to live well beyond limitations. In the case of cognitive decline, for example, the clinical psychologist for ageing operates between assessing mild cognitive impairment and dementia [9]. Here, the professionals educate people about cognitive impairment trajectories, teach about decline progression, facilitate individual responses to disease-induced changes, and value life history and the social/environmental contexts in the light of promoting well-being, quality of life, social inclusion, and dignity, even during the end-of-life phase

Promoting healthy lifestyles is paramount along the path of healthy ageing. In the prevention domain, the clinical psychologist of ageing can implement activities aimed at anticipating/minimising distress, discomfort, or disease onset. The activities start with empowering the ability to perceive the need to change life habits and plan the most personalised way to implement actions that can positively affect the life course. Psycho-education activities, for example, can be directed to people turning 50, with particular attention to those with health conditions known to represent risk factors for other diseases (e.g., hypertension, heart failure, diabetes)

Clinical psychologists working with ageing populations adopt multidimensional assessments, which capitalise on psycho-diagnostic tools capable of providing objective indications, where cut-offs and criteria are pre-determined and aid the professionals in disentangling complex situations. Assessments also investigate ageing people’s emotional and relational life aspects. Independently of the actual health conditions, multidimensional assessments capture risk factors (e.g., anxiety, depression, sleep disturbances, cardiovascular disorders) predisposing to cognitive decline, emerging disability, or mental illnesses. The assessments must interest all populations with chronic (e.g., hypertension, cardiovascular disorders) or degenerative (e.g., Parkinson’s disease) pathologies, which present an increased risk of developing mild or severe cognitive decline as well as several psychiatric conditions, such as depression. Thanks to the assessment, the psychologists can recognise and share the gist of the scene with all the parties. The sharing represents the initial step in composing a broader plan to modify expectations, attitudes, behaviour and language. In this vein, the clinical debrief/ final report is crucial. As defined by the Deontological Code of Italian Psychologists, the activities carried out by psychologists impact persons’ lives [10]. Independent of the working services, psychologists must consider the will of the persons they care for. In the ageing context, and even more importantly in the cases of degenerative disease, the person’s will should continuously be ascertained and the records updated.

The behavioural symptoms of dementia are paradigmatic, as they circularly affect and are caused by the interaction between patients and caring contexts, family included. To ease the symptom burden, the clinical psychologist of ageing highlights that symptoms are meaning bearers. The indications are based on assessing individual subjectivity, bodily functions, and context perception, as his work is focused on enhancing the capability to understand behaviours correctly. Assessment outcomes are unique in Italy: only a chartered psychologist can summarise and distill the meaning of such a complex scenario, whose final act is an articulated treaty shared with the interested parties [10].

Retirement, marital conflicts, bereavement, social isolation, hetero- and self-determined marginalisation, cognitive fatigue and physical exhaustion represent conditions where the person deserves a comprehensive working plan including understanding, care, and support. In all these conditions, when a person and their family get frail, they lose their grip on health and welfare status. Regardless of whether individuals can explicitly ask for help, the clinical psychologist of ageing represents a key figure. When the ominous events maturate on top of medical conditions, the psychologist can offer personalised ways to modulate personal habits due to sound theories and precise assessments and interventions.

Despite the clear need, in Italy, the clinical psychology of ageing is still an ancillary activity inside the services caring for ageing adults. The opinions expressed here represent the views of the *Clinical Psychology of Ageing* group, which is part of the Italian Association of Psychology, and aim to promote further discussion and advancements in mental health care for older adults.
