# Correction: The clinical psychology of ageing in Italy: Priorities for supporting the wellbeing of an ageing population

**DOI:** 10.1371/journal.pmen.0000414

**Published:** 2025-08-20

**Authors:** Giovanni Ottoboni, Elisa Di Rosa, Davide Maria Cammisuli, Maria Casagrande, Gianluca Castelnuovo, Ilaria Chirico, Anna Della Vedova, Isabella Giulia Franzoi, Mario Fulcheri, Antonella Granieri, Anna Pecchinenda, Luciano Peirone, Donatella Petretto, Maria Catena Quattropani, Alberto Sardella, Rabih Chattat

The eight author’s name is spelled incorrectly. The correct name is: Isabella Giulia Franzoi.

The ORCID iD for the eight author is missing. Please see the eight authors’ ORCID iDs here: 0000-0002-2455-330X (https://orcid.org/0000-0002-2455-330X).
